# Multi-scaling and mesoscopic structures

**DOI:** 10.1098/rsta.2009.0266

**Published:** 2010-03-13

**Authors:** E. K. H. Salje

**Affiliations:** Department of Earth Sciences, University of Cambridge, Downing Street, Cambridge CB2 3EQ, UK

**Keywords:** complexity, multi-scaling, microstructures, phase transitions

## Abstract

Multi-scaling and the systematic investigation of mesoscopic structures represent a field of fruitful cooperation in physics, chemistry, mineralogy and life sciences. The increasing miniaturization of devices as well as the emphasis of recent research on microstructures with length scales of a few nanometres lead to paradigm changes that may impact not only on our scientific understanding of fine-grained structures but also on the way we will develop device materials in the future. Here the role of interfaces becomes more important, and developments in areas such as ‘domain boundary engineering’ are evidence of this scientific evolution. In addition, nano-porous materials are particularly important in geology and in the development of artificial bones and ultra-light metals. Some of these developments are reviewed in this paper.

## 1. Introduction

To identify major future scientific developments, we need to understand that all predictions have the problem that they are concerned with the future. Few past developments in society or in science have been predicted correctly. It appears that most theories of the evolution of thought, in order to be consistent, require an extreme focus and a clear distinction between science and non-science. This paradigm still dominates our ideas about scientific development and often influences our academic ‘planning’. Empirically, science, and perhaps even the history of the human mind (but nothing concerning its societal impact), contains aspects that appear, so far, to follow trends that can be traced backwards over decades, with few singularities. Popular examples are the massive advance of computer power, which can be traced back to the discovery of the transistor, or the advances in biochemistry, which have as one origin the discovery of the DNA structure. The mechanisms that operate the Earth and some planets could not be understood without the concept of plate tectonics. Astrophysics needed a good understanding of the big bang. The time needed for such scientific developments is measured by the best part of a century, while shorter time scales may be, in reality, signs of lesser impact or even failure.

Computation, biochemistry, plate tectonics and astrophysics concern scientific endeavours with societal interest. Likewise, ‘evolution’, the ‘origin of life’ and the understanding of ‘complex systems’ are interrelated subjects that have fascinated society sufficiently that it appears safe to predict that much more progress may be expected in these fields. For example, it is not obvious that life is the necessary outcome of an increasing degree of complexity. Our understanding of complexity, even our ability to ask well-posed questions in its study, is not much developed and, for my part, I can see much room for progress here (for new developments, see Sherrington 2008). While complex systems are fundamentally different from more simple systems, as already stated by Wiener (1938), the multi-scaling of times and lengths or the role of entropy and entropy flow have not been understood conclusively (e.g. Bialek *et al.* 2001). One obstacle to research in this field is that the traditional distinctions between disciplines such as mathematics, physics, chemistry, life sciences and earth sciences, and even subjects placed in the humanities, have proved to be unhelpful. It is one of the positive current developments that such distinctions between fields of learning are blurred or even abolished. Progress is hence easier than previously. It is the purpose of this paper to speculate, with some trepidation, how some of the novel aspects of research on complex systems can profit from results in the field of condensed matter and how, potentially, these ideas can be generalized.

Multi-scaling, namely the treatment of problems on many length and time scales, is indeed one of the main developments in solid-state sciences, where small subject areas, such as mineralogy, probably advanced faster than, say, physics because minerals cannot be understood otherwise. Minerals often have very complicated crystal structures. These crystal structures are not, however, solely responsible for the macroscopic behaviour of minerals. An example concerning the elasticity of solids may illustrate this point. Over long time scales, a solid can behave like a fluid because its microstructures relax according to the laws of fluid mechanics while the bulk behaves perfectly elastically (Daraktchiev *et al.* 2007). This is the case for mantle minerals in geological processes (Faul & Jackson 2005). Short-time behaviour, on the other hand, is often dominated by avalanches and jerky ferroelastic behaviour, which dominate the behaviour in earthquakes. The same minerals will behave totally differently depending on the time and length scales of the experiment. The same types of behaviour are also found in applications of man-made materials such as shape memory alloys (SMAs) or elastomers (Adams & Warner 2008; Lashley *et al.* 2008; Salje *et al.* 2008). In the following, I will exemplify some of the more technical aspects of multi-scaling analysis and draw some wider conclusions at the end.

## 2. The mesoscopic length scale

The term ‘mesoscopic’ refers to ‘intermediate’ between long and short, and applies to microstructures to be seen in between the atomic and the macroscopic length scales. Very often the mesoscopic scale is located between 10 and 1000 nm, although the proper description implies that the *effective* rules for structural, dielectric, magnetic interactions, etc. are different from those of atoms and also different from those of a macroscopic body. Two aspects are important here: firstly, the fact that any finite body relaxes at the boundary (Houchmandzadeh *et al.* 1992; Conti & Salje 2001; Salje 2008); secondly, the fact that any heterogeneity is translationally invariant in an infinite body. A point defect or an interface can, in principle, move between positions of equal energy and (almost) equal entropy. Such a move will accommodate external agents such as strain, electric fields or magnetic fields. The elastic susceptibility (as the characteristic response function) is hence lowered, i.e. the sample is seen macroscopically as soft while no such softness exists in its proper crystal structure. Any calculation of macroscopic features needs, technically speaking, a certain degree of compactification and, often, the application of effective medium theories and the like (Milton 2002).

**Figure 1.**
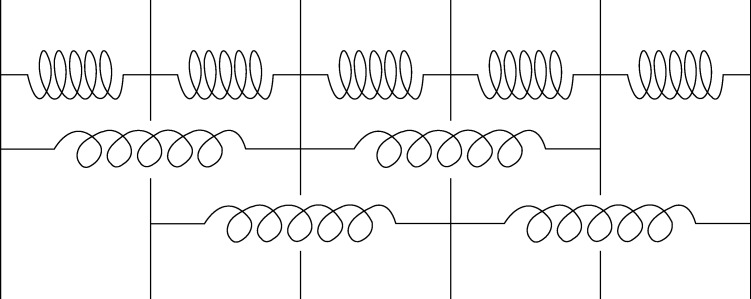
The simplest elastic chain involving surface relaxations. The outer layers are the surfaces, which have a reduced number of second nearest-neighbour interactions. We depict these as springs and generalize them to forces emanating from harmonic potentials so that the ‘spring constants’ can be positive or negative. Singularities occur when either the generalized nearest-neighbour spring constant vanishes or when the ratio of the spring constants between nearest and next nearest neighbours is −4. In these cases the surface relaxation invades the full space (Salje 2008).

**Figure 2.**
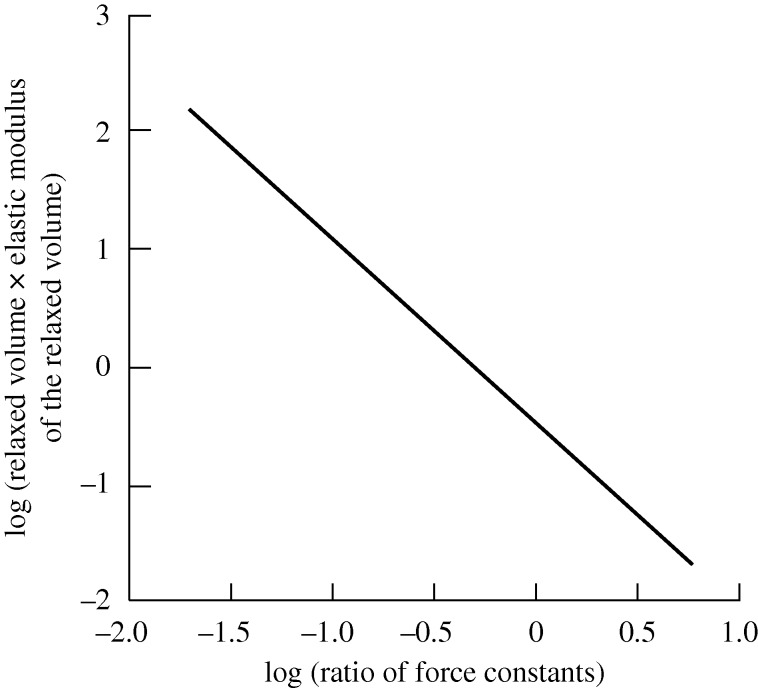
Power-law dependence of the relaxed surface volume with respect to the ratio of the force constants. The latter parameter is a measure of the temperature or pressure in real systems (Salje 2008). The integrated relaxation amplitude *δ*A near a surface increases when the critical point *t*_0_ is approached as 〈*δA*〉∼*t*^−3/2^.

Surface relaxations have been analysed and were found to be virtually independent of the concrete atomic potentials. All that matters is the fact that the second- and higher-order interactions are cut in any half space (figure 1). Any energy minimization of the remaining interactions leads to a series of differential equations with solutions of the type *ε*_*n*_=*λ*^*n*^*ε*_0_, where *ε*_*n*_ is the *n*th layer relaxation amplitude (strain), 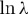 is the length scale of the relaxation and *ε*_0_ is the relaxation amplitude of the outermost layer. All structural relaxations have, thus, exponential envelopes and phase factors that are either +1 (ferroelastic), −1 (antiferroelastic), or exp(i*ϕ*) (incommensurate). The only variables are the amplitude of the relaxation, such as the amplitude of the first layer, and the length scale of the relaxation. Calculations using realistic potentials show that the amplitudes are around 2 per cent of the inter-atomic distances and the length scale is around 10 inter-atomic layers unless the system is close to instability, where this length scale diverges. This divergence leads to a collapse of the structure and ‘massive’ elastic softening. Such features are expected to dominate when solids undergo phase transitions while their grain size is on the nanometre scale. The singularity maintains a high degree of universality (e.g. a power-law decay of the elastic restoring force) so that this behaviour can be formulated as a ‘mesoscopic’ law of elasticity (or, in equivalent cases, electricity or magnetism), which is measured macroscopically and has, ultimately, atomic origins but operates only on its own specific length scale (figure 2).

**Figure 3.**
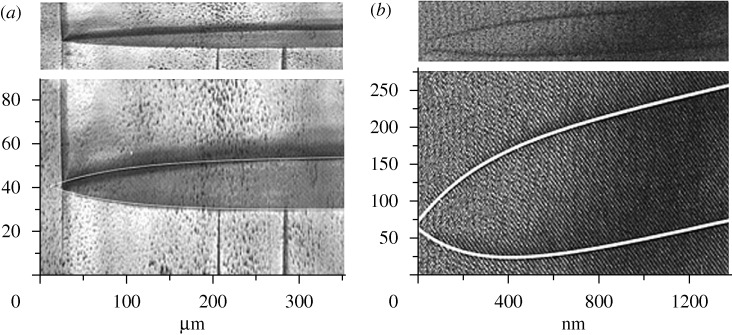
Macroscopic shape of ‘needle’ domains, which retract with little pinning force when subjected to external forces. The upper parts are the direct images; the aspect ratio of the vertical to horizontal axis has been increased in the lower parts to show the details of the trajectories. (After Salje *et al.* (1998) and Daraktchiev *et al.* (2007).)

The internal translational degree of freedom is maintained when external forces lead to the lateral movement of mesoscopic features such as twin boundaries or the retraction of pairs of twin boundaries that form geometrically needle- (or wedge-) shaped pairs (figure 3) (Salje & Ishibashi 1996; Salje *et al.* 1998). The movements of any such component leading to heat flux was thought to be impeded by lattice potential pinning, but lattice effects become (almost) irrelevant once the internal length scale of the twin wall, namely its width, becomes comparable with the lattice distances (Lee *et al.* 2006). The mesoscopic structure loses its memory of the underlying crystal structure in this case and moves without any significant interaction with the background structure. Its behaviour becomes fluid-like although the host lattice remains a solid. The only obstacles to such movements are defects that act over distances larger than the wall thickness and are hence on the same mesoscopic scale as the wall itself (Shilo *et al.* 2004). The result of the movement is a massive reduction of the macroscopically observed elastic response of the sample. The response is no longer elastic but contains aspects of fluid dynamics (for the free movement), avalanches and, potentially, elastic interactions with defects. Three typical examples are the pinned movement of LaAlO_3_ (Harrison *et al.*2004*a*,*b*), the free movement in pure SrTiO_3_ (Kityk *et al.* 2000) and the very weak pinning in KMnF_3_ and related structures (Salje & Zhang 2009*a*,*b*). Strong pinning by dislocations is observed in alloys in which also the thickness of the wall is much reduced. In this case the movement has nothing to do with either elastic or fluid behaviour but is dominated by avalanches and jerky behaviour (Bonnot *et al.* 2008). In a geological body any seismic response will average over all these features with a very low amplitude and relatively low frequency. Many of the singularities described above may not be visible under these circumstances, while others, such as relocation of walls via diffusion processes, may. Movements on a much longer time scale but on a similar length scale (Salje *et al.* 1998), such as those involved in upwelling in plate tectonics, will certainly experience material parameters that integrate all the long-term features and hence relate to much softer and fluid-like materials, which, under short-time-scale laboratory conditions, would appear as elastic solids with vastly different material parameters.

## 3. Heterogeneities and multi-ferroic behaviour

Ferroic phase transitions generate, under the appropriate boundary conditions, domain structures, which often dominate the macroscopic susceptibility of the material. Magnetic, ferroelectric and ferroelastic materials show very large values of their switchable magnetic moment, their polarization and their spontaneous strain, respectively, when domain structures change under external fields. The optimization of the domain structures to generate the largest possible susceptibility has been subject to much research and leads to the formulation of specific domain patterns as collective features. The stripe pattern with a high density of domain walls, the needle pattern with wedge-like needle domains, the junction pattern with many intersections of domain walls and the tweed pattern have all been extensively discussed in the literature (e.g. Daraktchiev *et al.* 2007). In non-ferroic, so-called co-elastic materials, the strain of the material may be significant but no switchable domain structures exist (Speer & Salje 1986; Salje *et al.* 1992; Jacobs *et al.*^2000^, ^2001^). This means that in this case all structural deformations occur exclusively in the bulk.

**Figure 4.**
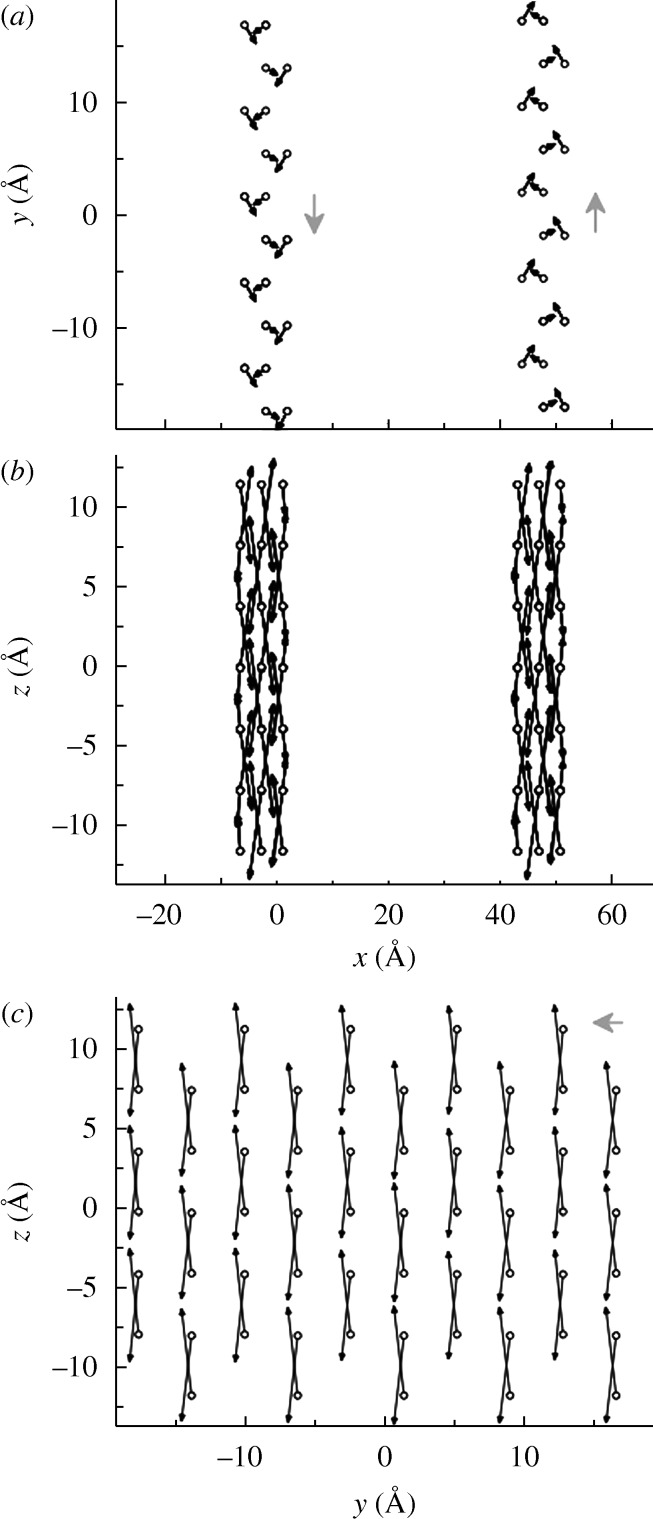
Dipole moments and atomic displacements of Ti inside twin walls of CaTiO_3_, as projected on to (*a*) the *xy*, (*b*) the *xz* and (*c*) the *yz* planes. The arrows indicate the Ti displacement from the centre of the corresponding O octahedron. Their sizes have been scaled by a factor of 300 with respect to the scale on the axes. (*c*) The twin wall plane. The grey arrows indicate the direction of the net polarization.

With the re-advent of a wider debate on multi-ferroic materials (Eerenstein *et al.* 2006; Cruz *et al.* 2007; Goncalves-Ferreira *et al.* 2008; Yu *et al.* 2008), the traditional issue of how one ferroic property can influence or even create another ferroic property becomes important. While a full analysis of experimental situations for bulk materials is still controversial, it has become clear that such multi-ferroic behaviour can originate from the internal domain wall structure and is, thus, a direct consequence of the mesoscopic structure, while the crystal structures matter less. The generic case is a ferroelastic material (say CaTiO_3_) with no other ferroic properties besides ferroelasticity (figure 4). When this material contains domains and ferroelastic domain walls, the domain walls themselves can be ferroelectric or ferrielectric—a property that does not exist in the bulk. Similarly, it may be possible to observe magnetic walls in ferroelastic or ferroelectric matrices. Related to such ‘local’ properties, one often observes that interfaces and walls are not simply the classic interpolation of bulk properties as seen in continuum theory. An example is served by zircon, which undergoes a local transformation into a glassy state when irradiated by radiogenic impurities. The damaged areas have a diameter of approximately 5 nm and are separated from the bulk by an interface of highly polymerized material (Farnan & Salje 2001). When the elastic susceptibility of this assembly is measured, one finds that the macroscopic sample shows a much stiffer elastic response than one would calculate in the Hashin–Shtrikman approximation of a two-phase mixture. The reason for the stiff behaviour is that the interface ‘protects’ the inner core of the damaged areas and prevents it from stronger compression. Hence the scaling of the elastic response is not that of the bulk proportions of the two phases (as usually assumed) but is strongly modified by the interfacial stress and, thus, shows the additional scaling of the interfaces (Salje 2006). This behaviour may be more widespread in other materials where the interfaces or domain walls modify the macroscopic behaviour significantly even when their volume proportion is modest. Static twin boundaries, on the other hand, seem not to generate elastic softening even though their internal density can be much less than that of the bulk (Goncalves-Ferreira *et al.* 2009).

These examples show that the internal structure of domain boundaries, interfaces, twin walls, etc. can have a significant effect on the macroscopic behaviour of material useful for engineering purposes. This effect can, in specific cases, even outperform the enhanced properties that come from the optimization of the domain structure. In this sense one would like to optimize the interfacial properties rather than the domain structure itself. Once this becomes possible we enter into an area in which ‘domain boundary engineering’ (Salje & Zhang 2009*a*,*b*) may provide answers where the more traditional ‘domain engineering’ fails or gives only inadequate solutions (Pertsev *et al.* 1996; Pertsev & Koukhar 2000; Yvry *et al.* 2007). The term ‘engineering’ implies the hope for future developments that will generate specific mesoscopic structures with desired properties that bulk materials cannot provide.

In the same way as mesoscopic structures were ‘invented’ when the translational symmetries of materials were broken, one can extrapolate this approach inside the mesoscopic structures themselves. Indeed, most mesoscopic structures are themselves made heterogeneous by doping when, for example, chemical defects are accumulated inside twin boundaries, dislocations, intercalations, etc. (Aird & Salje 1998). Chemical modifications of such structures are very common and have been extensively researched even when the doping was not intended and occurred by accident. Deliberate doping can lead to new compounds along the walls, while defects accumulate in the case of oxygen vacancies whereas the equivalent fluorite vacancies are less common or less significant for the pinning of twin walls. Finally, a very exciting new development for the self-pinning of twin walls and interfaces was postulated involving the spontaneous generation of dislocations by the moving interface. The role of the dislocations is then to exactly hinder this movement and this leads to aspects of shape memory behaviour (Groger *et al.* 2008). In SMAs equivalent studies of pinning processes of domain walls and their effect on aging have been clearly identified (Perez-Reche *et al.* 2004) and the role of dislocations during the first-order martensitic transition is now thought to be more pronounced than previously anticipated.

Another mechanism for a decreasing length scale of mesoscopic structures down to the atomic length scale appears as follows. Take a twin wall as a simple single mesocopic unit. This twin wall may now relate to twinning with respect to one specific structural deformation or, more precisely, one order parameter. Let us assume that this structural movement is simply a shear *Q* of the crystal structure. One twin is then sheared positively, the other negatively, and no shear occurs in the wall. The shear itself is often positively coupled with another deformation, e.g. the generation of a dipole moment *P*. A positive coupling energy *λQ*^2^*P*^2^ with *λ*≫0 prevents the secondary feature from occurring in the bulk. Inside the wall, however, the shear becomes zero and the coupling vanishes. The secondary parameter can now become finite (*P* non-zero). This second parameter has a choice: it can be positive (e.g. *P*>0) or negative (*P*<0). Both solutions are degenerate in the original problem. We may now assume that both solutions exist with the same probability. The issue is then: what are the features if these two solutions connect topologically in the wall? The answer is that they again form a wall but now with a reduced dimensionality. While the original wall was a sheet with 2+*ε* dimensions, the new wall reduces this dimension by one, i.e. the new walls are topologically lines (or strings), which have a small but finite surface tension. In magnetism this effect was predicted, and the strings are then called Bloch lines. The same construction can now be used to reduce the strings to points (which may be called Bloch points), which are in reality blobs with a finite diameter (akin to quantum dots), albeit theoretically smaller than one atomic diameter. This example shows that mesoscopic features can both lead to very significant macroscopic responses to external fields and also reduce their size and dimensionality, finally going back to an atomic or even smaller length scale. Theoretically at least, such structures could be read macroscopically by appropriate fields so that the equivalent storage density, if a device could ever been made, would exceed those of current constructions by approximately four orders of magnitude (thickness of the chip 1 mm, only two-dimensional features with characteristic distance of 10 nm; one-dimensional memory elements would increase the density even further).

## 4. Complexity, multi-scaling and statistical mechanics

While multi-scaling is now commonly used in the treatment of mesoscopic structures in solids and liquids, we have certainly not fully understood its full potential, in particular not in the life sciences. Most theories simply minimize the energy when the stability of a mesoscopic structure is calculated, while entropic terms are virtually always ignored. This is clearly incorrect when the relevant length scale approaches inter-atomic dimensions. Both the dynamical excitations and the topological order/disorder will contribute to the entropy with very different temperature dependences. Dynamical contributions to the entropy may be hard to detect at high temperatures, while they should modify the quantum saturation of the structural order parameter (Salje *et al.* 1991). The configurational entropy is best seen in specific heat measurements when spontaneous changes, such as avalanches, modify the latent heat of a phase transition. Finally, the mesoscopic structures will lead to a rescaling of the length and time not only in a geometrical, electrical and magnetic sense but also in their electronic structure. It is only very recently that surface relaxations have been analysed in order to detect changes of their band structure and electronic properties (Pruneda *et al.* 2007).

Extremely large surface areas exist in porous materials, which have been widely researched: in the earth sciences, because they have simple fingerprints for seismic signals; in medicine, because artificial bones for transplants require a network of holes for the insertion of organic matter, collagen, etc.; and in engineering of ultra-light metals. Porous materials are unstable when the percolation of solid matrix vanishes. It is a typical problem of mesoscopic science to investigate the limit of elastic stability. It has been argued that elastic instabilities can occur as a continuous transition (where the product phase is virtual) and a strongly first-order transition leading to the disintegration of the sample. The generic phase diagrams of such massive elastic instabilities can then be divided into two classes. Consider the primary control parameter such as the porosity or any other related physical quantities. The secondary control parameter is stress in the same way as in ferroelastic materials. The relevant thermodynamic quantities related to these two control parameters are the elastic moduli (or Young’s modulus, the bulk and the shear moduli and, to a much lesser extent, Poisson’s ratio). The parameter space for the porosity is [0,1], and the bulk modulus changes between *κ*_0_ and 0.

The first type of phase diagram holds for most porous minerals and materials investigated so far and is based on a model of random holes and percolation of these holes in an elastically stiff matrix (Fritsch *et al.* 2007). This singularity is contained in the case of non-interacting spheres where the bulk modulus decays almost linearly from the solid value to the transition point near a porosity of *P*=0.5. In the second scenario, we consider the phase diagram as a two-phase mixing problem with randomly distributed voids with strong interactions or elongated shapes, and a solid phase with bulk modulus *κ*_0_. In first approximation the effective elastic bulk modulus is given by the upper bound of the Hashin–Shtrikman limit, which decays continuously until *P*=1. In a more detailed, self-consistent model (Fritsch *et al.* 2009), it was shown that the assembly remains stable until this porosity limit is reached, albeit with smaller values of *κ*. This scenario applies for bone materials, mesoporous silica and corals, and is of greatest importance for medical applications, while the former scenario is typical in most geological materials such as shales and cements.

While the development of complex structures and structural features on a mesoscopic length scale may have been best understood in condensed matter and fluid mechanics, this would not be the natural choice for a start. In bio- and organic chemistry the natural length scale is usually much bigger than in inorganic solids. Complexity is reached at a somewhat bigger length scale, while the internal mechanisms operate on the same atomic scale that we know well in inorganic systems. Protein folding is an example for a very large unit that requires the consideration of enthalpic and entropic properties for the assessment of their statistical mechanics and kinetics. Entropy and entropy flow are also crucial parameters for the modelling of other complex systems in both biology and game theory, while this aspect has been explored to a much lesser extent, so far, in inorganic solids. However, there appears very little evidence that evolutionary tendencies are directly related to complexity at a molecular level. As an example, it appears that protein-coded genes are almost as complex in sea anemones as in humans (Technau 2008). The distinction may well be related to the small RNAs that protect the genome from transposes and show a high degree of evolutionary flexibility (Grimson *et al.* 2008). The key for such behaviour seems to lie in the fact that even very simple systems, such as illustrated above in the case of simple harmonic chains with competing springs, lead already to bifurcations and dynamic complexity. This argument has already been made some time ago by May & Oster (1976) and has lost none of its significance. Complexity may not, in the sense as discussed here, lead to novel behaviour, but when complexity is combined with multi-scaling, it may well produce phenomena that go far beyond our current expectations.
